# Clinically Important Drug–Drug Interactions Between Antiarrhythmic Drugs and Anticoagulants

**DOI:** 10.19102/icrm.2019.100304

**Published:** 2019-03-15

**Authors:** Kaja M. Konieczny, Paul Dorian

**Affiliations:** ^1^Division of Cardiology, Department of Medicine, St. Michael’s Hospital, University of Toronto, Toronto, ON, Canada

**Keywords:** Antiarrhythmic drug, anticoagulant, drug interaction

## Abstract

Until the last decade, vitamin K antagonists (VKAs) were the only agents available for oral anticoagulation. Although effective and accessible, their use was complicated by a narrow therapeutic window, the need for regular monitoring of the international normalized ratio, and an associated susceptibility to interactions with both food and numerous medications. Furthermore, the onset of action was delayed, often requiring bridging with intravenous agents. In more recent years, we have enjoyed the development of nonvitamin-K-dependent, direct oral anticoagulants (DOACs), which either directly inhibit the activity of factor IIa (eg, dabigatran) or factor Xa (eg, rivaroxaban, apixaban, edoxaban). These medications boast a more rapid onset of action, predictable pharmacokinetics, wider therapeutic window, and equal or superior safety profiles. Although these medications appear to have fewer drug–drug interactions than VKAs, their interactions remain of clinical importance, particularly in one of the largest populations requiring anticoagulation: patients with atrial fibrillation. These patients are rarely on single medications, with the majority of them requiring some form of rate or rhythm control due to their arrhythmia. Unfortunately, data on interactions between DOACs and antiarrhythmic medications, despite their common coadministration, remain limited. Here, we summarize the interactions between antiarrhythmics and VKAs and review existing knowledge regarding their interactions with DOACs.

## Introduction

Until the last decade, vitamin K antagonists (VKAs), including warfarin, acenocoumarol, and phenprocoumon, were the only agents available for oral anticoagulation. Although effective and accessible, their use was complicated by a narrow therapeutic window, the need for regular monitoring of the international normalized ratio (INR), and an associated susceptibility to interactions with both food and numerous medications. Furthermore, the onset of action was delayed, often requiring bridging with intravenous agents, due to the time required to suppress the synthesis of vitamin-K-dependent clotting factors.

In more recent years, we have enjoyed the development of nonvitamin-K-dependent direct oral anticoagulants (DOACs), which either directly inhibit the activity of factor IIa (eg, dabigatran) or factor Xa (eg, rivaroxaban, apixaban, edoxaban). These medications demonstrate a more rapid onset of action, predictable pharmacokinetics, wider therapeutic window, and equal or superior safety profile. However, although these medications appear to have fewer drug–drug interactions than VKAs do, their interactions still remain of clinical importance, particularly in one of the largest populations requiring anticoagulation: patients with atrial fibrillation. These patients are rarely on single medications, with the majority of them requiring some form of rate or rhythm control for their arrhythmia. Unfortunately, data on the interactions between DOACs and antiarrhythmic drugs (AADs), despite their common coadministration, remain limited. Here, we will summarize the interactions between AADs and VKAs and review existing knowledge on their interactions with DOACs.

## Basic principles of drug interactions

The introduction of a drug into a living organism results in a complex interplay of processes. Unfortunately, the nature of this complex interaction between the drug and multiple factors is such that many constituent events are inherently variable, potentially compromising the desired result of administration. This may be further influenced by the coadministration of other medications.^1,2^

In the determination of interactions between AADs and both VKAs and DOACs, the most relevant metabolic enzyme system is the cytochrome P450 (CYP) superfamily, which is abundantly expressed in hepatic tissue and which is responsible for most of the metabolism of up to 50% of drugs. A given substance may be a substrate, inducer, or inhibitor of one or more CYP isoforms. Furthermore, a drug may induce or inhibit CYP enzymes not involved in its own metabolism. In general, the CYP-mediated reactions provide a means of eliminating an active drug, but, occasionally, they may also produce active metabolites or activate a prodrug.^3–6^ Typically, CYP-mediated interactions tend to occur independently of the timing of drug administration; thus, temporally spacing the administration of involved medications has little value.

Of equal importance is drug transportation across cellular membranes. P-glycoprotein (P-gp) is a cellular transport protein involved in both cellular uptake into target cells and the elimination of drugs and their metabolites, functioning as an efflux transporter.^7^ It is extensively expressed in enterocytes, hepatocytes, renal tubular cells, and some endothelial cells. Many additional transport proteins play a role in clinical pharmacokinetics, but none as mischievously as P-gp. Most commonly, P-gp plays a role in drug efflux; therefore, the inhibition of its activity leads to elevated drug levels. Often, P-gp–mediated interactions can be minimized by altering the timing of affected drugs, such as taking them two hours or more apart.

## Antiarrhythmic medications

### Classification

AADs constitute a large family of medications used to suppress cardiac dysrhythmias. Their actions are exerted via the inhibition of various ion channels and pumps as well as the alteration of autonomic function.

Class I AADs share the common feature of sodium-channel blockade, but they exhibit variable kinetics and effects on potassium channels, prompting subdivision into three classes.^2,8^ Class Ia agents including quinidine, procainamide, and disopyramide share the common feature of moderate rates of onset of phase 0 depression as well as additional potassium channel blockade. They increase both action potential duration (APD) and the effective refractory period (ERP) of depolarizing cells. In contrast, class Ic drugs, such as flecainide and propafenone, exhibit slow-onset phase 0 depression; thus, their effects accumulate at increasing rates. Furthermore, they produce no effect on either APD or ERP. Class Ib agents affect predominantly ventricular myocardial cells. They exhibit rapid onset and offset of phase 0 depression and reduce both ADP and ERP.

Class II AADs, more commonly called β-blockers, constitute a large and heterogeneous group of medications, all of which inhibit β-adrenergic receptors.^9^ This review will focus on interactions involving commonly used agents, including bisoprolol, metoprolol, carvedilol, and propranolol.

Class III AADs share the property of potassium-channel blockade, but many of these medications have other, additional mechanisms of action.^2,8^ The most commonly used class III agents are amiodarone, dronedarone, sotalol, ibutilide, and dofetilide.

Class IV AADs block calcium channels and include verapamil and diltiazem.^10^ Dihydropyridine calcium channel blockers will not be discussed in this review.

Digoxin, a cardiac glycoside, is not classified within the Vaughan–Williams system due to its distinct mechanism of action.^11^ It inhibits sodium-potassium adenosine triphosphatase, leading to the intracellular accumulation of sodium. This prompts an exchange of intracellular calcium via the sodium–calcium exchange system, resulting in intracellular calcium accumulation. Digoxin also exerts sympatholytic and vagotonic effects through mechanisms that are not well-understood at this time but which are known to produce some degree of atrioventricular nodal blockade.

### Pharmacokinetics

AADs constitute a pharmocokinetically heterogeneous group of medications not amenable to generalization. The majority of these drugs are CYP substrates, and many are CYP inhibitors **(Table 1)**.^2^

Drug–drug interactions involving antiarrhythmics have been extensively investigated, but interactions with more novel agents are not as well-characterized as those of more traditional medications.

#### Class I antiarrhythmics

Quinidine is a substrate of CYP3A4 and an inhibitor of the CYPs 3A4 and 2D6.^2,12^ It is actively secreted by P-gp in the renal tubule, whose activity it strongly inhibits. For medications that rely on P-gp for elimination, such as digoxin, coadministration with quinidine leads to marked increases in digoxin concentrations. Quinidine has a strong affinity for P-gp, and its own pharmacokinetics do not appear to be as susceptible to marked fluxes in P-gp activity.

Several agents, namely calcium channel blockers, have been noted to interact with quinidine, independently of pharmacokinetic effects. The mechanisms of these interactions are not clear.

Procainamide undergoes predominantly renal clearance of both the parent drug and its active metabolite, N-acetyl procainamide. It is not a substrate, inducer, or inhibitor of any CYP isoforms or of P-gp.^2^

Disopyramide is a substrate of CYP3A4 but does not appear to alter the activity of any CYP isoforms.^2,12^

Lidocaine is a substrate of CYPs 1A2, 2B6, and 2D6 and exerts an inhibitory effect specifically on CYP1A2.^2,12^ It undergoes predominantly hepatic metabolism, whereas metabolites are largely renally cleared.

Mexiletine is a CYP2D6 and CYP1A2 substrate, inhibiting the latter.^2,12^ It is extensively metabolized in the liver, with 8% to 15% of the drug undergoing renal excretion unchanged. The metabolites have minimal clinical activity.

Flecainide is metabolized in the liver by CYP2D6 (which it inhibits) to a number of metabolites, none of which have significant antiarrhythmic activity.^2,12^ The remaining unchanged drug, representing approximately one-third of the administered dose, is renally cleared, along with its drug metabolites.

Propafenone is almost completely metabolized by CYPs 1A2, 2D6, and 3A4 and inhibits CYP2D6.^2,12^ Furthermore, it has two active metabolites, conferring a substantial potential for drug interactions. Propafenone is not a substrate of P-gp but does inhibit its activity.

#### Class II antiarrhythmics

Propranolol, a nonselective β-blocker, undergoes extensive hepatic metabolism with involvement of the CYPs 2D6, 1A2, and 2C19.^12^ There is minimal renal clearance of the parent drug, but the metabolites are renally cleared. One of the metabolites is a weak inhibitor of CYP2D6, although the clinical significance is questionable. Propranolol is both a substrate and an inhibitor of P-gp.

Bisoprolol, one of the newer cardioselective β-blockers, undergoes a moderate degree of hepatic metabolism predominantly with involvement of CYP3A4.^12^ CYP2D6 has been found to play a minor metabolic role. The remainder of the drug, representing approximately one-half of the administered dose, is excreted unchanged in the urine. Clinical data suggest that bisoprolol may be a substrate of P-gp, but this has not been definitively proven.^13^ Coadministration with propafenone leads to elevated bisoprolol levels, presumably due to CYP2D6 inhibition.

Metoprolol, also cardioselective, is a substrate of CYP2D6, with 80% of the drug undergoing metabolism via this route.^12^ Only 5% of the drug is renally cleared unchanged. It does not appear to inhibit any CYP isoform, nor is it a substrate or inhibitor of P-gp.^13^

Carvedilol is a nonselective β-blocker and an α_1_-antagonist. It is almost completely metabolized by the CYPs 2D6 and 2C9, with minor involvement of the CYPs 3A4, 1A1, 1A2, 2C19, and 2E1.^12^ Many metabolites are generated, one of which is a potent β-blocker and three of which are weak vasodilators. All of the metabolites are renally cleared. Carvedilol is a potent inhibitor of P-gp but not a substrate of it. Coadministration with amiodarone in a heart failure population led to increased plasma carvedilol levels via inhibition of the drug’s metabolism. Coadministration with digoxin does not alter carvedilol levels, but digoxin levels are increased due to P-gp inhibition.^13^

#### Class III antiarrhythmics

Amiodarone is metabolized to its predominant and active metabolite desethylamiodaron by the CYPs 3A4 and 2C8, while inhibiting the CYPs 1A2, 2D6, 2C9, and 3A4.^2,12^ It has been implicated in many drug interactions of clinical importance. Although not a P-gp substrate, oral amiodarone is a potent inhibitor of P-gp.

Dronedarone is metabolized by CYP3A4 to its less active *N*-debutyl metabolite, exerting inhibitory effects on both the CYPs 3A4 and (to a lesser extent) 2D6.^12^ It is also an inhibitor of P-gp, but not its substrate.

Sotalol is not metabolized and is instead renally cleared unchanged.^2,12^ It is neither a substrate nor an inhibitor of P-gp.

Ibutilide undergoes predominantly hepatic metabolism, which requires further characterization, but it occurs independently of the CYPs 3A4 and 2D6.^2^ To our knowledge, to date, no formal drug–drug testing has been carried out; however, β-blockers, calcium channel blockers, and digoxin have been coadministered with no measurable impact on the pharmacokinetics of ibutilide.

Dofetilide is predominantly renally cleared via glomerular filtration and active secretion by the cationic transport system.^2^ This occurs independently of P-gp.

#### Class IV antiarrhythmics

Verapamil exerts its antiarrhythmic effects via the cardioselective inhibition of L-type calcium channels, although it has some affinity for a variety of other calcium channels.^10^ It undergoes extensive hepatic metabolism, predominantly involving the CYPs 3A4, 3A5, and 2C8, with minor metabolism via the CYPs 1A2, 2C9, 2C19, 2D6, and 2E1.^12^ It is widely recognized as a potent inhibitor of CYP3A4. Verapamil is both a substrate and an inhibitor of P-gp.

Diltiazem also blocks L-type calcium channels and is a substrate of the CYPs 3A4, 2C8, 2C9, and 2C19.^10,12^ It is a well-recognized inhibitor of CYP3A4 and may have inhibitory effects on CYP2D6. It is both a substrate and an inhibitor of P-gp.

Both verapamil and diltiazem have many documented drug interactions.^12^

## Anticoagulants

### Classification

Warfarin and other VKAs inhibit the synthesis of factors II, VII, IX, and X. Dabigatran directly inhibits the activity of thrombin, whereas rivaroxaban, apixaban, and edoxaban directly inhibit the activity of factor X.

### Pharmacokinetics and interactions with antiarrhythmic medications

#### Coumarin (vitamin-K-dependent) anticoagulants

Warfarin, along with both acenocoumarol and phenprocoumon, is a vitamin K reductase inhibitor.^12^ The reduced form of vitamin K, KH_2_, is a cofactor in the carboxylation and activation of factors II, VII, IX, and X as well as in proteins C, S, and Z. Although the latter three proteins have anticoagulant properties, the net effect of KH_2_ depletion is an anticoagulant one.^14^

Warfarin is administered as a racemic mixture of two active enantiomers, *R*-warfarin and *S*-warfarin, and exhibits near-total albumin binding in the plasma. Although the metabolites are renally excreted, there is very little renal excretion of the active drug, which instead is metabolized stereoselectively and regioselectively **(Table 2)**. *S*-warfarin, the more potent enantiomer of the two, undergoes extensive metabolism by CYP2C9. As a result, CYP2C9 polymorphisms may produce varying rates of warfarin clearance. This is of particular importance at the initiation of drug therapy, prior to the establishment of a steady state. *R*-warfarin undergoes metabolism by the CYPs 1A2 and 3A4 predominantly as well as the CYPs 1A1, 2C8, 2C9, 2C18, and 2C19 to a lesser extent.

Acenocoumarol and phenprocoumon, like warfarin, are largely protein-bound and undergo metabolism by CYP2C9. There is virtually no renal excretion of the active drug. Metabolites, all believed to be inactive, are renally cleared.

Although the metabolism of coumarin is almost exclusively hepatic, with no significant renal clearance of active compounds, patients with renal disease have been found to require lower coumarin doses. Chronic kidney disease can alter the nonrenal clearance of drugs; in this case, this is felt to be a consequence of decreased CYP2C9 activity in the context of renal insufficiency.^15^

Drug interactions with coumarin-based anticoagulants are numerous and typically explained by the induction or inhibition of the CYPs 2C9, 1A2, and 3A4 **(Table 3)**.^12^ Among the antiarrhythmics, quinidine, propafenone, and amiodarone can all inhibit coumarin metabolism and cause marked potentiation of the anticoagulant effect. Although “antiarrhythmics” are considered to be agents that may reduce the INR, none of the above AADs have been clinically proven to do so to date. Of note, coadministration with dronedarone produces clinically negligible elevation in *S*-warfarin levels, with an insignificant effect on the INR.

#### Dabigatran

Dabigatran, administered as its oral prodrug, dabigatran exilate, is cleaved into its active form by a serum esterase.^12^ It acts as a competitive, reversible direct thrombin inhibitor, inhibiting free, fibrin-bound, and clot-bound thrombin. Oral bioavailability is 3% to 7% when the capsule is swallowed intact, but it may double this amount if the capsule is breached. Peak plasma concentrations occur at two hours after administration but may be delayed by coadministration with food. Eighty percent of the active drug is renally excreted, 6% is fecally excreted, and the remainder undergoes metabolism by serum esterases and microsomal carboxylesterases. Four metabolites are produced, each of which has a direct inhibitory effect on thrombin, albeit with < 10% of the potency of the parent compound. The CYP system has not been found to play any role in the metabolism of dabigatran, nor is the drug an inducer of these enzymes. It is, however, a P-gp substrate **(Table 2)**. Coadministration with inhibitors of P-gp results in elevated plasma levels of dabigatran. P-gp inducers produce the opposite effect. In healthy volunteers, the half-life is 12 hours to 14 hours.

Given the extensive renal clearance of this drug, renal impairment prolongs the half-life and increases dabigatran levels. Dose adjustments are recommended when creatinine clearance is less than 50 mL/min, whereas drug administration is advised against when it is less than 30 mL/min.

Drug interactions with dabigatran, occurring as a result of P-gp inhibition and producing elevated dabigatran levels, could be anticipated with use of the following antiarrhythmics: quinidine, propafenone, propranolol, bisoprolol, oral amiodarone, dronedarone, verapamil, and diltiazem **(Table 3)**.^12,16^ The possibility has not been yet tested in all these agents, however. When coadministered with oral amiodarone, amiodarone levels were unchanged, while dabigatran levels were increased by 50% to 60%. With dronedarone coadministration, dabigatran levels increased markedly, and the combination is currently contraindicated.^16^ Coadminstration with verapamil can lead to elevated dabigatran levels, but the degree of elevation depends on the formulation of verapamil as well as the timing of administration. The most pronounced effect was observed with immediate-release verapamil taken one hour prior to dabigatran, in which case, the anticoagulant levels were nearly tripled. Extended-release formulations increased levels by 150% to 180%. It is recommended that dabigatran dosing be reduced to 150 mg once daily in both of these scenarios.^12^ There was no meaningful interaction observed when verapamil was given two hours after dabigatran.

Coadminstration of dabigatran and quinidine, a potent P-gp inhibitor, is contraindicated.^12^

#### Rivaroxaban

Rivaroxaban is a competitive and irreversible direct factor Xa inhibitor of both free and clot-bound Xa, which functions independently of antithrombin III.^12^ Oral bioavailability approaches 100% when taken with food, and peak plasma concentrations are achieved at two hours to four hours postdose. Approximately one-third of the orally administered drug undergoes renal clearance unchanged. This is predominantly the result of active renal secretion by P-gp, among other transport proteins. The remainder undergoes hepatic transformation into inactive metabolites by the CYPs 3A4 (18%), 3A5 (minimal), and 2J2 (14%), which are subsequently renally and fecally excreted to similar extents **(Table 2)**. In total, renal clearance accounts for two-thirds of drug elimination. None of the metabolites have been found, however, to have a clinically relevant effect. In healthy volunteers, the half-life is five hours to nine hours, increasing to 11 hours to 13 hours in the elderly.

In the context of renal impairment, plasma concentrations of rivaroxaban increase by approximately 1.5-fold across a broad range of insufficiency. As a result, dose reduction is recommended in patients with an estimated glomerular filtration rate (eGFR) of less than 50 mL/min but generally not recommended if the eGFR is less than 15 mL/min. Of note, the Efficacy and Safety Study of Rivaroxaban with Warfarin for the Prevention of Stroke and Noncentral Nervous System Systemic Embolism in Patients with Nonvalvular Atrial Fibrillation (ROCKET-AF) trial excluded patients with an eGFR value of less than 30 mL/min at the index visit.

In patients with mild hepatic impairment, only small changes in the pharmacokinetics were noted. Changes are more pronounced with moderate impairment, resulting in elevated plasma concentrations and prolonged elimination. In this instance, the increase in drug exposure is due to reductions in both hepatic and renal clearance, the latter occurring independently of creatinine clearance and possibly representing a manifestation of reduced tubular secretion of the parent drug.^17^

Owing to the involvement of the CYPs 3A4 and 2J2 in the oxidation of rivaroxaban, researchers have sought to assess the effects of coadministration with drugs known to interfere with these pathways. Rivaroxaban has not been found to be an inducer or inhibitor of any major CYP isoforms. Coadministration with strong inhibitors of both CYP3A4 and P-gp have led to 2.5-fold increases in rivaroxaban exposure. Strong inducers of both CYP3A4 and P-gp had the opposite effects.

Drug interactions could be expected between rivaroxaban and the CYP3A4 inhibitors oral amiodarone, dronedarone, quinidine, and diltiazem (the latter three of which are also P-gp inhibitors) as well as the P-gp inhibitors propafenone, propranolol, carvedilol, and verapamil. The degree of interaction has not been quantified in the literature to date **(Table 3)**.^16^ Notably, the administration of rivaroxaban with moderately potent CYP3A4 inhibitors produces no clinically relevant adverse effects. There is no mutual interaction between rivaroxaban and digoxin. It has been recommended that coadministration of rivaroxaban and dronedarone be avoided.^16^

#### Apixaban

Like rivaroxaban, apixaban is a direct inhibitor of both free and clot-bound factor Xa, but it acts in a highly selective and reversible manner.^12,18^ Oral bioavailability approximates 50%, with peak plasma levels reached at three hours to 3.5 hours after administration. Apixaban is a substrate of P-gp. Only 25% of the active drug is renally cleared, with the remainder undergoing metabolism by predominantly CYP3A4. The CYPs 1A2 and 2J2 produce minor metabolites, with the CYPs 2C8, 2C9, and 2C19 playing an even smaller role in the drug’s metabolism **(Table 2)**. Apixaban has a clinically insignificant inhibitory effect on CYP2C19. It does not inhibit P-gp, nor does it induce the activity of pharmacokinetically relevant enzymes. Half of the parent drug is excreted unchanged, reducing the potential for clinically important drug–drug interactions. There are no active metabolites. In healthy volunteers, the half-life is nine hours to 14 hours.

In patients with renal impairment, plasma apixaban concentrations are increased. This is more pronounced in cases of more severe impairment, but maximally by 1.5-fold. Renal impairment has no measurable impact on the drug’s anti-Xa effect. In North America, dose adjustment is recommended when two of the following three criteria are met: age ≥ 80 years, body weight ≤ 60 kg, and creatinine ≥ 133 μmol/L (1.5 mg/dL). In Europe, dose reduction is advised for an eGRF of between 15 mL/min and 29 mL/min. Apixaban use is not recommended in patients with an eGFR of less than 15 mL/min.

In studies of patients with mild to moderate hepatic impairment, no effect on the pharmacokinetics was observed.^12^

When cadministered with strong inhibitors of both P-gp and CYP3A4, apixaban concentrations increased nearly twofold. Conversely, the opposite effect is seen with coadministration with strong inducers of both P-gp and CYP3A4, with plasma concentrations falling to half of baseline values.

Drug interactions can be anticipated when apixaban is coadministered with CYP3A4 or P-gp inhibitors including quinidine, propafenone, carvedilol, propranolol, oral amiodarone, dronedarone, diltiazem, and verapamil **(Table 3)**. In existing clinical trials, no significant interactions were found between apixaban and atenolol or digoxin.^12,16^ A minimal, but clinically insignificant, effect is noted with the coadministration of apixaban and diltiazem, with 1.3-fold to 1.4-fold increases in apixaban levels noted. No dose adjustment is recommended in this instance.^12^

#### Edoxaban

Edoxaban is a reversible direct factor Xa inhibitor that is transported across the intestinal wall by P-gp.^12^ Oral administration results in 62% bioavailability, with peak plasma levels achieved at one hour to two hours. Anticoagulant effects are rapid and dose-dependent, with a direct concentration–effect relationship. Fifty percent is renally cleared, with the remainder undergoing metabolism or fecal clearance. Although many edoxaban metabolites have been found to have some anticoagulant activity, their low concentration results in insignificant clinical effects. Of note, there is minimal metabolism of edoxaban by both CYP3A4 (< 10%) and CES1 (< 10%), resulting in the production of minute quantities of two minor metabolites **(Table 2)**. Edoxaban is not an inducer of major CYP enzymes or of P-gp, but it does have a weak inhibitory effect on the latter. In healthy volunteers, the half-life is 10 hours to 14 hours.

In patients with renal impairment, exposure to both active edoxaban and its metabolites increases. With rising degrees of renal impairment, metabolic clearance plays a proportionately greater role. Dose reduction is recommended in patients with an eGFR of less than 50 mL/min but is not generally recommended if the eGFR is less than 15 mL/min.

In contrast, hepatic impairment does not significantly impact peak or total edoxaban exposure, although it has only been assessed in patients with mild to moderate dysfunction (Child–Pugh classes I and II).

Given the above pharmacokinetics, drug interaction studies investigating the effects of CYP3A4 and P-gp inhibition on coadministered edoxaban have been performed.^12^ Briefly, CYP3A4 inhibitors increase both peak and total levels of edoxaban, as do P-gp inhibitors, but neither does so by more than twofold. CYP3A4 and P-gp inducers have the opposite effect.

Interactions between edoxaban and a variety of antiarrhythmics can be expected given the similar pharmacokinetics of edoxaban and apixaban. Coadministration with digoxin, a noninducing, noninhibitory P-gp substrate, produced no interactions. P-gp inhibitors, including quinidine, oral amiodarone, dronedarone, and verapamil, have been coadministerred with edoxaban, resulting in elevated edoxaban levels **(Table 3)**.^18^ This finding was most pronounced with quinidine and dronedarone, both of which are also CYP3A4 inhibitors, where dose reduction of edoxaban is recommended by some sources.^18^ Similar interactions can be expected with propafenone, carvedilol, propranolol, and diltiazem, but these have not been quantified in the published literature. Similarly, the effect of dosage timing modification has not been investigated.

## Conclusions

The introduction of DOACs as an alternative to VKAs in patients requiring anticoagulation has, in many instances, simplified the management of patients with concomitant arrhythmias; however, one must remain cognizant of the existing potential for drug–drug interactions. Certain combinations of medications require increased monitoring, whereas others require dosage adjustment. In a few instances, specific combinations are contraindicated.

In general, patients on anticoagulants should be followed carefully for bleeding complications, particularly when additional risk factors for bleeding exist such as marginal renal function or low body weight.

In patients receiving antiarrhythmics, their efficacy should be regularly evaluated and their use continued only if they are found to be effective and necessary.

## Figures and Tables

**Table 1:** Pharmacokinetics of Antiarrhythmic Drugs

Antiarrhythmic Class	Agent	CYP Substrate	P-gp Substrate	Enzymes/Transporters Inhibited
Class Ia	Quinidine	3A4	Yes	3A4, 2D6, P-gp
	Procainamide	No	No	None known
	Disopyramide	3A4	No	None known
Class Ib	Lidocaine	1A2, 2B6, 2D6	No	1A2
	Mexiletine	2D6, 1A2	No	1A2
Class Ic	Flecainide	2D6	No	2D6
	Propafenone	1A2, 2D6, 3A4	No	2D6
Class II	Propranolol	2D6, 1A2, 2C19	Yes	P-gp, weakly 2D6
	Bisoprolol	3A4 (minor: 2D6)	Possibly	None known
	Metoprolol	2D6	No	None known
	Carvedilol	2D6, 2C9 (minor: 3A4, 1A1, 1A2, 2C19, 2E1)	No	P-gp
Class III	Amiodarone	3A4, 2C8	No	1A2, 2D6, 2C9, 3A4, P-gp
	Dronedarone	3A4	No	3A4, 2D6, P-gp
	Sotalol	No	No	None known
	Ibutilide	No	No	None known
	Dofetilide	Insignificant	No	None known
Class IV	Verapamil	3A4, 3A5, 2C8 (minor: 1A2, 2C9, 2C19, 2D6, 2E1)	Yes	3A4, P-gp
	Diltiazem	3A4, 2C8, 2C9, 2C19	Yes	3A4, possibly 2D6, P-gp

CYP: cytochrome P-450; P-gp: P-glycoprotein.

**Table 2:** Pharmacokinetics of the Anticoagulants

Classification	Agent	CYP Substrate	P-gp Substrate
Coumarin anticoagulant (VKA)	Warfarin	*S*-warfarin: 2C9	No
		*R*-warfarin: 1A2,	
		3A4 (minor: 1A1, 2C8, 2C9, 2C18, 2C19)	
	Acenocoumarol	2C9	No
	Phenprocoumon	2C9	No
Direct thrombin inhibitor	Dabigatran	No	Yes
Direct factor Xa inhibitor	Rivaroxaban	3A4, 2J9 (minor: 3A5)	Yes
	Apixaban	3A4, 1A2, 2J2 (minor: 2C8, 2C9, 2C19)	Yes
	Edoxaban	3A4	Yes

CYP: cytochrome P-450; P-gp: P-glycoprotein; VKA: vitamin K antagonist.

**Table 3:** Drug–Drug Interactions Involving Anticoagulants and Antiarrhythmics

Antiarrhythmic Drug	Mechanism of Interaction		Warfarin	Dabigatran	Rivaroxaban	Apixaban	Edoxaban
Quinidine	•	Warfarin: 3A4 inhibition	Potentiated anticoagulant effect	53% increase	Extent of increase unknown	No data yet	77% increase
	•	DOACs: P-gp competition					
Flecainide	•	None anticipated	None known	No data yet	No data yet	No data yet	No data yet
Propafenone	•	Warfarin: 3A4 inhibition	Potentiated anticoagulant effect	No data yet	No data yet	No data yet	No data yet
Propranolol	•	Warfarin: 1A2 inhibition	Potentiated anticoagulant effect	No data yet	No data yet	No data yet	No data yet
Carvedilol	•	DOACs: P-gp competition	No effect	No data yet	No data yet	No data yet	No data yet
Metoprolol	•	None anticipated	None known	No data yet	No data yet	No data yet	No data yet
Amiodarone	•	Warfarin: 3A4, 1A2, 2C9 inhibition	Potentiated anticoagulant effect	12%−60% increase	Minor effect	No data yet	40% increase
	•	DOACs: P-gp competition					
Dronedarone	•	Warfarin and DOAC: P-gp competition, 3A4 inhibition	Potentiated anticoagulant effect	70%–100% increase, combination contraindicated	Moderate effect, combination contraindicated	No data yet, caution advised	85% increase; consider dose adjustment or different DOAC
Sotalol	•	None anticipated	None known	No data yet	No data yet	No data yet	No data yet
Dofetilide	•	None anticipated	None known	No data yet	No data yet	No data yet	No data yet
Verapamil	•	Warfarin: 3A4 inhibition	Potentiated anticoagulant effect	12%–180% increase (if taken simultaneously); consider dose adjustment or different DOAC	No effect	No data yet	53% increase
	•	DOACs: P-gp competition, weak 3A4 inhibition					
Diltiazem	•	Warfarin: 3A4 inhibition	Potentiated anticoagulant effect	No effect	No effect	40% increase	No data yet
	•	DOACs: P-gp competition, weak 3A4 inhibition					

DOAC: direct oral anticoagulant; CYP: cytochrome P-450; P-gp: P-glycoprotein.
